# Congruency of intervening events and self-induced action influence prediction of final results

**DOI:** 10.1007/s00221-020-05735-9

**Published:** 2020-01-29

**Authors:** Tsukasa Kimura, Jun’ichi Katayama

**Affiliations:** 1 The Institute of Scientific and Industrial Research (ISIR), Osaka University, Ibaraki, 567-0047 Japan; 2 Department of Psychological Science, Kwansei Gakuin University, Nishinomiya, Japan; 3 Center for Applied Psychological Science (CAPS), Kwansei Gakuin University, Nishinomiya, 662-8501 Japan

**Keywords:** Expectation, Sense of agency, Sensory attenuation, ERP

## Abstract

Predicting self-induced stimuli is easier than predicting externally produced ones and the amplitude of event-related brain potentials (ERP) elicited by self-induced stimuli is smaller than that elicited by externally produced ones. Previous studies reported that these phenomena occurred strong when stimuli were presented immediately after self-induced action. To be able to adapt to changes, however, it is necessary to predict not only an event that follows a self-induced action but also a subsequent final result. We investigated whether congruency among self-induced actions, intervening events, and final results influences the processing of final results. The congruency of an intervening event with self-induced action was task-irrelevant information for the required response to a final result. The results showed that the P1 amplitude elicited by the final result (i.e., somatosensory stimulus) when an intervening event was congruent with self-induced action was smaller than other elicited amplitudes. This suggests that the congruency of an intervening event and self-induced action may facilitate prediction of a final result, even when this congruency is irrelevant to the ongoing task.

## Introduction

In everyday life, our environments are rich in sensory stimuli that provide us with sensory information. In addition, results generated by our self-induced actions also provide sensory information. For example, knocking on a door generates a sound and provides auditory information. It is reported that predicting a self-induced event becomes easier than predicting an externally produced event. One explanation for this phenomenon is an internal forward model based on planning of self-induced action, copying of motor commands, and sensory feedback resulting from the self-induced action (e.g., Blakemore et al. 1999; Wolpert 1997; Wolpert et al. 1995). In this explanation, it is thought that a motor command copy is sent as input to the internal forward model when we plan a self-induced action. Moreover, it is conceivable that this information is compared with the sensory feedback generated by the self-induced action. It is thought that predicting a self-induced event is easier than predicting an externally produced event because self-induced action uses this internal system. However, some previous studies have proposed other theories. Sensory attenuation studies require the performance of a self-induced action (e.g., pressing a button). In this situation, it is possible that self-induced action includes allocating attention to the action itself, and that sensory attenuation might occur due to the distribution of attention between the action and the result (e.g., Horváth 2015; Hughes and Waszak 2011). Moreover, participants might acquire awareness of the relationship between the self-induced action and its result through experiencing the trials. Therefore, it is possible that this relationship influences the processing of the result and leads to the preactivation of the result of one’s actions (e.g., Horváth 2015; Roussel et al. 2013). No conclusion has been reached yet regarding what internal process influences the prediction; however, it is thought that predicting a self-induced event is easier than predicting an externally produced event in this theory.

The intensity with which one predicts a self-induced event is thought to be related to a sense of agency, i.e., feeling that our actions are controlled by us and that events are generated by our actions (e.g., Friston 2012; Sato and Yasuda 2005). One of the parameters of the sense of agency is temporal contiguity between self-induced actions and events. We feel that an event was generated by self-induced action when the time interval between the self-induced action and the event was short (e.g., Farrer et al. 2008; Sato and Yasuda 2005). Moreover, perceptions of sensory stimuli generated by self-induced action decrease in strength when events accord with our predictions. For example, we do not feel ticklish when we tickle ourselves (e.g., Blakemore et al. 1999; Blakemore et al. 2000). According to the internal forward model, this phenomenon, called “sensory attenuation”, results when a sensory stimulus corresponds with a prediction generated by self-induced action. It is thought that sensory attenuation and sense of agency are intertwined, for example, sensory attenuation becomes weaker as the temporal delay between self-induced action and event increases, and so does sense of agency (e.g., van Elk et al. 2014; Sato and Yasuda 2005). On the other hand, some previous studies reported that sensory attenuation and sense of agency are independent phenomena. For example, some studies reported that sensory attenuation is influenced not by sense of agency but by congruency between self-induced action and results of that action (e.g., Dewey and Knoblich 2014; Timm et al. 2016). Therefore, it is possible that sensory attenuation can be explained by correspondence of the result and congruency between self-induced action and the result without sense of agency, whether or not a sense of agency occurs.

Sensory attenuation is reported not only in behavioral studies but also in electrophysiological studies. For example, N1/P1 amplitudes of the event-related brain potential (ERP) elicited by self-induced stimuli are smaller than those elicited by externally produced ones (e.g., Horváth 2015). In a somatosensory study, sensory attenuation of the somatosensory-evoked potential (SEP) thought to originate in SII correlates with behavioral sensory attenuation (e.g., Palmer et al. 2016). Moreover, the amplitude of ERP about 100 ms after a stimulus decreases when the temporal delay between pressing a key (i.e., self-induced action) and a subsequent sound (i.e., event) is short and fixed (e.g., Bäß et al. 2008; Martikainen et al. 2005; Schafer and Marcus 1973). These results suggest that temporal contiguity between self-induced actions and results influences the prediction of results at the level of electrophysiological activity and decreases the processing load involved in perceiving the results.

These studies provide evidence regarding how we adapt to changes in our environment. To live adaptively, however, it is unlikely that we will need to predict only events that occur immediately after self-induced actions. For example, when we throw an apple up in the air, it is conceivable that the apple might float there. However, we can predict that the apple will fall down due to gravity and will return to our hand after that. In other words, it is possible to predict not only the event that will occur immediately after the self-induced action, but also an event that will occur after this intervening event.

However, it remains unclear whether relationships among self-induced actions, intervening events, and final results influence the processing of the final results. In everyday life, we need to predict the distant future to live adaptively. By examining the relationship among self-induced actions, intervening events, and final results, it is possible to examine prediction in everyday life that is not limited to self-induced action and what happens immediately afterwards. Previous studies reported that congruency of results with self-induced action facilitates prediction of the results (e.g., Bäß et al. 2008; Horváth 2015; Hughes et al. 2013; Waszak et al. 2012). Moreover, this congruency influences the processing of evaluation of results. Evaluating a result generated by self-induced action is important if we are to optimize our behavior. Feedback-related negativity/reward positivity (FRN/RewP) is elicited by bad/good results generated by action (e.g., Proudfit 2015; Ullsperger et al. 2014). The amplitude of FRN/RewP decreases with a delay between an action and its result, and it is thought that this delay impairs the processing involved in evaluating the result generated by the action (e.g., Peterburs et al. 2016; Weinberg et al. 2012). A recent study reported that sequential intervening events promote the prediction of results generated by self-induced action and do not impair the amplitude of FRN/RewP (e.g., Kimura and Kimura 2016). Although this study focused on the effect of temporal congruency of sequential intervening events on prediction of results, other studies have reported an effect of spatial congruency on such prediction (e.g., Doherty et al. 2005: Kimura and Katayama 2017a). These studies suggested that the congruency of sequential intervening events influences the prediction of a subsequent result. Therefore, it is possible that congruency among a self-induced action, an intervening event, and the final result facilitates prediction of the final result, even if the final result occurs long after the self-induced action.

To test this hypothesis, we manipulated whether intervening events and final results were congruent with self-induced actions and compared resulting ERPs as an index of sensory attenuation. It is known that a preceding task-irrelevant visual stimulus influences the spatial, temporal, and stimulus type of prediction of subsequent somatosensory stimulus and ERPs elicited by that stimulus (e.g., Kimura and Katayama 2015, 2017a, b, 2018). In the present study, we composed experimental conditions using the paradigm of these studies. At first, participants were required to press a left- or right-hand button (i.e., self-induced action) to initiate trials. After the key was pressed, a white circle (i.e., intervening event) flashed three times on either the left or the right side of the desk. Finally, somatosensory stimuli (i.e., final result) were presented with a high probability (80%) of being applied to one wrist and a low probability (20%) of being applied to the opposite wrist, and participants were asked to perform a simple reaction time task with their foot in response to all somatosensory stimuli. In all trials, high-probability somatosensory stimuli were presented on the same side as the white circles, and low-probability somatosensory stimuli were presented on the opposite side. In other words, spatial congruency existed between the intervening event and final result in all trials, and this congruency was relevant information for the simple reaction time task in response to the somatosensory stimulus, because high-probability somatosensory stimuli were presented on the same side as the white circles. By contrast, congruency between self-induced action and the intervening event was manipulated among conditions. White circles flashed invariably on the side where the button was pressed in the fixed congruent condition (fixed congruent event), whereas in the random condition, these circles flashed on the side where the button was pressed for half of the trials (random congruent event) and on the opposite side for the other half of the trials (random non-congruent event). In other words, spatial congruency of the intervening event and self-induced action existed invariably in the fixed congruent condition, whereas this congruency was uncertain in the random condition. In addition, this congruency was irrelevant information for the simple reaction time task of responding to the somatosensory stimulus because high-probability somatosensory stimuli were presented not on the side where the key was pressed but on the side where the white circles were presented in all trials. Therefore, the difference among trial types was only the presence or absence of spatial congruency between self-induced action and intervening event as task-irrelevant information. Previous studies reported that an external somatosensory stimulus without self-induced action elicited ERPs in a condition similar to the present experimental condition, and that a task-irrelevant visual stimulus influenced these ERPs (e.g., Kimura and Katayama 2015, 2017a, b, 2018). Therefore, it is possible to examine the effect of congruency between a self-induced action and an intervening event on a response for somatosensory stimulus (i.e., result) by regarding a visual stimulus as an intervening event.

To examine the effect of differences in congruency on prediction, we compared P1 amplitudes elicited by somatosensory stimuli (i.e., final result). Previous studies reported that ERP amplitude about 100 ms after a stimulus was attenuated when sensory attenuation occurred due to an auditory or visual stimulus (e.g., Horváth 2015). It remains unclear whether ERP amplitude about 100 ms after a somatosensory stimulus is definitely decreased by self-induced action, although many behavioral data and phenomena have been reported. In a few studies, sensory attenuation of somatosensory was reported to occur in less than 100 ms (MEG: Hesse et al. 2009; ERP: Palmer et al. 2016). Moreover, P1 had a clear peak in this study as electrophysiological activity of less than 100 ms, and it occurred even in research using a similar experimental paradigm (e.g., Kimura and Katayama 2015, 2017a). Unlike a study using an auditory stimulus, the studies using somatosensory stimuli have not reported that the attenuation for specified ERP amplitude, but in this study, we followed previous studies and used P1 as electrophysiological activity of less than 100 ms. P1 is a mid-latency component of SEP and reflects not only the physical characteristics of a stimulus but also the consciousness (e.g., Eimer and Forster 2003; Kida et al. 2004; Schubert et al. 2006). P1 is elicited about 100 ms after a somatosensory stimulus and is generated from a secondary somatosensory area (e.g., Allison et al. 1989a, b, 1992). Previous studies reported that an external somatosensory stimulus without self-induced action elicited P1 in a condition similar to the present experimental condition. Additionally, P1 amplitude did not differ between high-probability stimuli and low-probability stimuli in these studies (e.g., Kimura and Katayama 2015, a). Moreover, a few studies reported that the amplitude of P1 elicited by self-induced action decreased (e.g., Palmer et al., 2016). Therefore, this paradigm certainly elicits the P1 component, and it is able to examine sensory attenuation by comparing high-probability stimuli among conditions. We predicted that if congruency of an intervening event and self-induced action facilitates the prediction of a final result, P1 amplitude would decrease in the fixed congruent event.

Moreover, we compared amplitudes of P3 elicited by somatosensory stimuli to confirm the effect for deviation from prediction and for an intervening event. P3 is elicited by an unexpected stimulus, e.g., low-probability stimulus, and this amplitude reflects the intensity of a deviation from expectation (e.g., Donchin 1981; Duncan-Johnson and Donchin 1977; Katayama and Polich 1996). In this study, the probability of the final result was manipulated by congruency between intervening event and final result. The final result was presented with high probability at the same side as the intervening event or with low probability at the opposite side. If the participants ignored the intervening event, the effect of probability would not occur and P3 would not be elicited by any stimulus. On the other hand, if participants used the congruency between intervening event and final result and if the congruency of an intervening event and self-induced action also influences the processing of a deviation from expectation, P3 would be elicited by a low-probability somatosensory stimulus, and this influence might increase the amplitude for the fixed congruent event.

Finally, we compared amplitudes of contingent negative variation (CNV) elicited between the third white circle and the somatosensory stimulus among trials to confirm that the time prediction did not differ among conditions. CNV is related to temporal expectations prompted by a prior stimulus regarding a subsequent stimulus (e.g., Walter et al. 1964). In a similar experiment using presentation of lights and somatosensory stimuli, CNV was elicited between the light presentation and subsequent somatosensory stimuli (e.g., Kimura and Katayama 2018). Therefore, if participants are able to predict the timing of somatosensory stimuli in all trials, CNV would not differ between trials.

Through these comparisons of ERPs among congruent events, we examined whether congruency between self-induced action and intervening events influences expectations of final results. The difference among congruent events was only spatial congruency of the intervening event with self-induced action (or lack of such congruency) as task-irrelevant information. Therefore, if congruency influences the expectation of a final result, this expectation would occur more easily in the fixed congruent event than in the other conditions.

## Method

### Participants

Fourteen undergraduate and graduate students (9 females, 5 males; 18–25 years of age) participated in the experiment. One participant was left-handed and the others were right-handed, according to their self-report. All participants had normal or corrected-to-normal vision. This experiment was approved by the Kwansei Gakuin University (KGU) Research Ethics Review Board under the KGU Regulations for Research with Human Participants. Written informed consent was obtained from all participants, and their rights as experimental subjects were protected.

### Stimuli and procedure

**Fig. 1:**
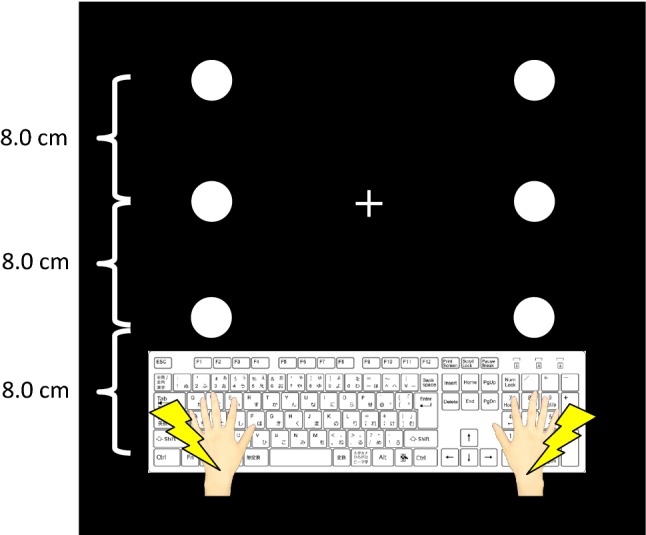
The positions of stimulus and fixation presentation. The white circles indicate the positions of visual stimuli (i.e., intervening event), the thunder icons indicate the positions of somatosensory stimuli (i.e., final event), and the white cross indicates the position of the fixation point

Figure 1 shows the positioning of the somatosensory stimuli, visual stimuli and fixation cross. Somatosensory stimuli were generated by an electrical stimulus generator (Nihon Koden Corporation, SEN-7203) and were adjusted by electric isolators (Nihon Koden Corporation, SS-203J). To present somatosensory stimuli to participants, Ag/AgCl electrodes (diameter of 1.0 cm) were put on their forearms. The anode electrodes were placed on the participants' wrists, and the cathode electrodes were placed 3.0 cm from the anodes toward the elbow. The stimuli were single block pulses of 0.2 ms in duration. The intensities were three times as high as the threshold for each participant (never causing pain). The absolute threshold was measured by six iterations of the up-and-down method per participant. The average intensity of the stimuli across all participants was 3.4 mA. These stimuli were presented to the right or left wrist at each trial.

Visual stimuli, a fixation cross and a background black color were projected on the desk via LCD projector (EPSON, EB-1430WT). The stimuli were white circles and the diameter of each circle was 3.8 cm. These circles were placed at equal distances on the desk (8.0 cm each) and the distances of the leftmost and rightmost circles from left and right hands of the participants were also the same. The duration of stimuli was 200 ms. The white fixation cross was placed near the center circle and was presented until the end of the block.

Each trial was started by pressing a left-hand button (the F button on the keyboard) with the participant’s left index finger or pressing a right-hand button (the J button on the keyboard) with the right index finger. After the key was pressed, three visual stimuli and one subsequent somatosensory stimulus were presented. The interval (SOA) from the onset of the first visual stimulus to the second visual stimulus, from the second visual stimulus to the third visual stimulus, and from the third visual stimulus to the somatosensory stimulus was invariably set to 1000 ms. The interval between trials was either 1000 or 1200 ms at random with equal probability.

**Fig. 2:**
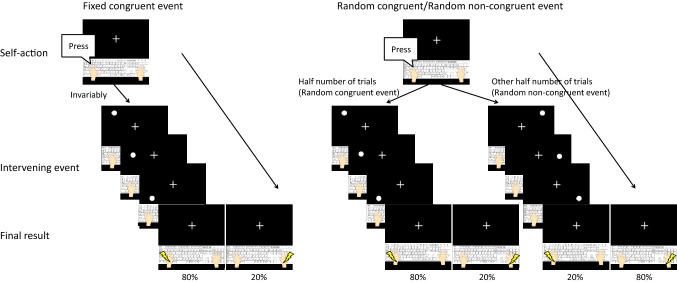
The procedures of fixed congruent events, random congruent events, and random non-congruent events

The two conditions were distinguished by congruency or incongruency between the key pressed and visual stimuli, and this congruency was administered in separate blocks. Figure 2 shows the procedure for each condition. In the fixed congruent condition, the visual stimuli were presented sequentially toward the wrist on the same side on which the key was pressed (i.e., if the participant pressed the left button, the visual stimuli were presented on the left side), and the subsequent somatosensory stimulus was presented with high probability (80%) to that wrist or low probability (20%) to the opposite wrist (fixed congruent event). In the random congruent condition, the visual stimuli were presented sequentially toward the wrist on the same side on which the key was pressed for half of the trials (random congruent event). For the other half of the trials, the visual stimuli were presented on the opposite side (random non-congruent event). However, the subsequent somatosensory stimulus was presented with a high probability (80%) to the wrist on that side or with a low probability (20%) to the wrist on the opposite side. The order of the random congruent and non-congruent events was completely randomized in the block. In summary, the congruency of the location of the visual stimuli and the location of the button pressing was different in the two conditions; however, the congruency of the location of the somatosensory stimulus and the location of the visual stimuli was the same in both conditions. Each block was composed of 44 trials (in 32 trials, somatosensory stimuli were presented on the same side as the visual stimuli, in eight trials, they were presented on the opposite side, and no somatosensory stimulus was presented in four catch trials), which took approximately 4 min. Four blocks were presented for the fixed congruent condition. To equalize the number of the fixed congruent trials from the point of view of congruency of the final result with the intervening event, eight blocks were presented for the random congruent condition. The interval between blocks was 2 min, and the participants rested for 5 min after finishing the six blocks. The order of conditions was randomized between participants.

In the experimental room, the participants were asked to sit at a desk and to place their arms on the desk 32.0 cm apart. In addition, they were required to gaze at the fixation point, to control their eye movements, and not to move their eyes and bodies more than necessary in each condition. Moreover, the participants were instructed to press a left- or right-hand key with equal probability to start the trials, and to respond using a foot pedal with the left (or right) foot when the somatosensory stimuli were presented, and not to respond when somatosensory stimuli were not presented (i.e., the catch trials). In half of the blocks, the participants used the left or right foot; in the other half, they changed the side and used the opposite foot in each condition. Thus, the relation of the side of the key press to the foot response was not confounding in this experiment. Finally, participants were told before each block that there was a high probability that the somatosensory stimuli would be presented not to the same side on which the key was pressed, but to the same side on which the white circles were presented. In other words, the congruency of the presentation of somatosensory stimuli (i.e., final result) and the presentation of the white circles (i.e., intervening event) was relevant information for the task, but the congruency of the presentation of the white circles and the side on which the key was pressed (self-induced action) was irrelevant information.

### Recording and analyses

EEG data were recorded by BrainAmp (Brain Products, Germany) and an electrode cap (Easycap GmbH, Germany) using Ag/AgCl electrodes at 30 sites (Fp1, Fp2, F7, F3, Fz, F4, F8, FT7, FC3, FCz, FC4, FT8, T7, C3, Cz, C4, T8, TP7, CP3, CPz, CP4, TP8, P7, P3, Pz, P4, P8, O1, Oz, O2) according to the modified 10–20 System. In addition, electrodes were also placed on both earlobes (A1 and A2). The reference electrode was on the tip of the nose, and the ground electrode site was AFz. The data from all channels were recorded using Brain Vision Recorder software (Version 2.0, Brain Products, Germany). The electrode impedances were kept below 5 kΩ. A bandpass filter of 0.1–200 Hz was used at recording. The sampling rate was 1000 Hz.

To analyze the EEG data, the EEGLAB toolbox (Delorme and Makeig 2004) and ERPLAB toolbox (Lopez-Calderon and Luck 2014) on MATLAB (MathWorks Inc) were used. The data were digitally low-pass filtered at 30 Hz (6 dB/octave) using an IIR Butterworth analog simulation filter. Artifacts derived from eye movements and eye blinks were rejected using an automatic EEG artifact detector based on the joint use of spatial and temporal features (ADJUST) of the EEGLAB toolbox (Mognon et al. 2011). To extract P1 and P3, the EEG epoch was set at 1000 ms (including a 200 ms prestimulus baseline). The epoch in which the EEG signal variation exceeded ± 100 μV was excluded from averaging. Additionally, trials with RTs shorter than 200 ms or longer than 1500 ms and trials with incorrect responses were discarded from the analysis. After artifact rejection, the numbers of remaining trials ranged from 116 to 128 (0–10% of trials were rejected) for the high-probability stimulus and 29–32 (0–9.4% rejected) for the low-probability stimulus in fixed congruent events, 115–128 (0–10.2% rejected) for the high-probability stimulus and 30–32 (0–6.3% rejected) for the low-probability stimulus in random congruent events, and 112–128 (0–12.5% rejected) for the high-probability stimulus and 29–32 (0–9.4% rejected) for the low-probability stimulus in random non-congruent events. The time range of P1 was defined as 80–120 ms and P3 was defined as 220–360 ms. These time ranges were decided by peak latencies of grand averaged waves for all conditions used in the analysis.

In addition, to investigate CNV, the EEG epoch was set at 1200 ms (the baseline was a − 200 to 0 ms prestimulus of the third visual stimulus, and the onset of the somatosensory stimulus occurred at 1000 ms). The epoch in which the EEG signal variation exceeded ± 100 μV and trials with errors were excluded from averaging. After artifact rejection, the numbers of remaining trials were 141–160 (0–11.9% rejected) for the fixed congruent events, 141–160 (0–11.9% rejected) for the random congruent events and 143–160 (0–10.6% rejected) for the random non-congruent events. The mean CNV amplitude was obtained from a latency window of 500–1000 ms. The appropriate latency window was defined based on observation of the resultant ERP waveforms.

At first, to check for bias due to pressing a key to start trials, the number of key presses using the left vs. right buttons was compared by paired *t* test in both conditions. The effect size was calculated by computing Cohen’s *d* (Cohen 1988). Next, a two-way repeated-measures analysis of variance (ANOVA) of reaction times (RTs) in response to the electrical stimuli was conducted with the three congruent events [fixed congruent event, random congruent event, and random non-congruent event) × two stimulus probabilities (high probability (80%) and low probability (20%)]. Moreover, the mean amplitude of P1 elicited by high-probability stimuli at Pz, where the P1 was elicited at maximum amplitude, was assessed with a one-way repeated-measures ANOVA (three congruent events). Furthermore, the P3 mean amplitude was assessed with a three-way repeated-measures ANOVA [3 congruent events × 2 stimulus probabilities × 3 electrodes (Fz, Cz and Pz)]. These electrodes were chosen to check the distribution of P3 amplitude at the midline. In addition, the mean CNV amplitudes at FCz, where the CNV was elicited at maximum amplitude, were compared between trials by a one-way repeated-measures ANOVA (three congruent events). These ANOVAs were conducted by applying Greenhouse–Geisser corrections to the degrees of freedom (Greenhouse and Geisser 1959) when Mauchly's sphericity test was significant. The effect sizes have been indicated in terms of partial eta squared (*η*). Post hoc comparisons were made using Shaffer's modified sequentially rejective multiple test procedure, which extends Bonferroni *t* tests in a stepwise fashion (Shaffer 1986). The significance level was set at *p* < 0.05 for all statistical analyses.

## Results

### Behavioral data

The check for bias due to pressing a key to start the trial revealed no significant different between the left and right keys in the fixed congruent condition [left: 89.93; right: 86.07; *t*(13) = 1.11, *p* > 0.10] and random congruent condition [left: 176.43; right: 175.57; *t*(13) = 0.15, *p* > 0.10]. Therefore, the trials involving pressing the left key and pressing the right key were integrated. Table 1 shows the mean RTs of all participants. The ANOVA showed that the RTs of responses to the low-probability stimulus were longer than those for the high-probability stimulus (*F*(1, 13) = 13.11, *p* = 0.003, *η* = 0.50). However, the main effect of congruent event (*F*(2, 26) = 1.62, *p* > 0.10) and their interaction (*F*(2, 26) = 2.80, *p* = 0.09) was not significant.

**Table 1:** Mean RTs (ms) for somatosensory stimuli and standard errors of RTs in each congruent event

	Fixed congruent event	Random congruent event	Random incongruent event
High probability	346 (20.87)	349 (20.10)	341 (18.64)
Low probability	380 (25.28)	369 (23.93)	365 (24.74)

### Electrophysiological data

#### P1

**Fig. 3:**
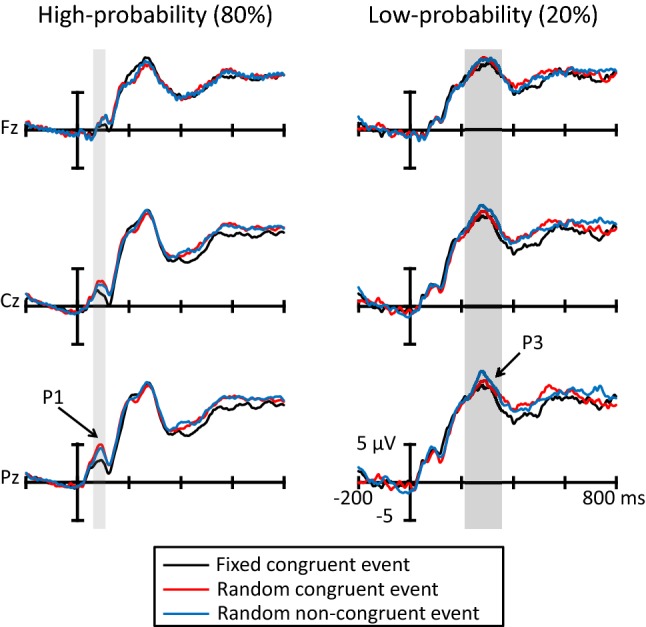
Grand average ERP waveforms for each trial at the Fz, Cz, and Pz electrode sites (*N* = 14). The light gray area denotes the time range of P1 (80–120 ms), and the dark gray area denotes the time range of P3 (220–360 ms)

Figure 3 shows the grand averages for ERPs elicited by somatosensory stimuli during the fixed congruent event (black lines), random congruent event (red lines) and the random non-congruent event (blue lines) from Fz, Cz, and Pz. The first positive deflection showed peak latency at about 80 ms (P1) and the second positive deflection showed peak latency at about 290 ms (P3).

**Fig. 4:**
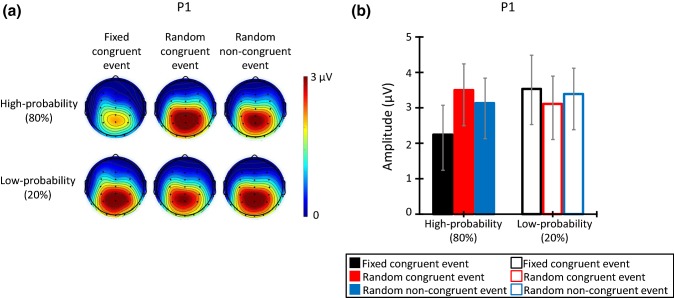
**a** The topographic map of the P1 time range (80–120 ms), and **b** mean P1 amplitude for each congruent event (*N* = 14). The error bars indicate the standard errors (SEs) of the means across participants

Figure 4 illustrates (a) the topographic map at the time range of P1 (80–120 ms), and (b) the P1 mean amplitude in all trials. The ANOVA for the mean amplitude of P1 elicited by the high-probability stimulus revealed a significant main effect of congruent event (*F*(2, 26) = 8.37, *p* = 0.005, *ε* = 0.72, *η* = 0.39). Post hoc comparisons indicated that the high-probability stimulus in the fixed congruent event elicited smaller P1 amplitudes than the high-probability stimulus in the random congruent and random non-congruent event (*p*s < 0.05). These results revealed that P1 amplitudes elicited by the high-probability stimulus in the fixed congruent event were weaker than the others.

#### P3

**Fig. 5:**
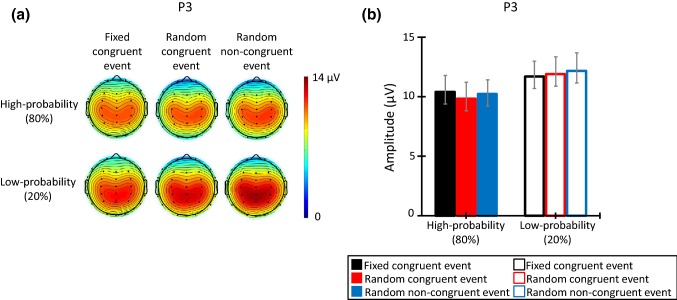
**a** The topographic map of the P3 time range (220–360 ms), and **b** mean P3 amplitude for each congruent event (*N* = 14). The error bars indicate the standard errors (SEs) of the means across participants

Figure 5 shows (a) the topographic map at the time range of P3 (220–360 ms), and (b) the P3 mean amplitude in all trials. The ANOVA showed that the P3 amplitudes elicited by the low-probability stimuli were larger than those elicited by the high-probability stimuli (*F*(1, 13) = 39.88, *p* < 0.001, *η* = 0.75). Moreover, the main effect of electrodes was significant (*F*(2, 26) = 8.53, *p* = 0.006, *ε* = 0.67, *η* = 0.40). Post hoc comparisons revealed that the electrodes of Cz and Pz elicited larger P3 amplitudes than Fz (*p*s < 0.05). However, the main effect of congruent event (*F*(2, 26) = 0.14, *p* > 0.10) and all interactions was not significant (congruent event and stimulus probabilities: *F*(2, 26) = 0.84, *p* > 0.10; congruent event and electrodes: *F*(4, 52) = 0.26, *p* > 0.10; stimulus probabilities and electrodes: *F*(2, 26) = 2.80, *p* = 0.08; trials, stimulus probabilities and electrodes: *F*(4, 52) = 0.21, *p* > 0.10).

#### CNV

**Fig. 6:**
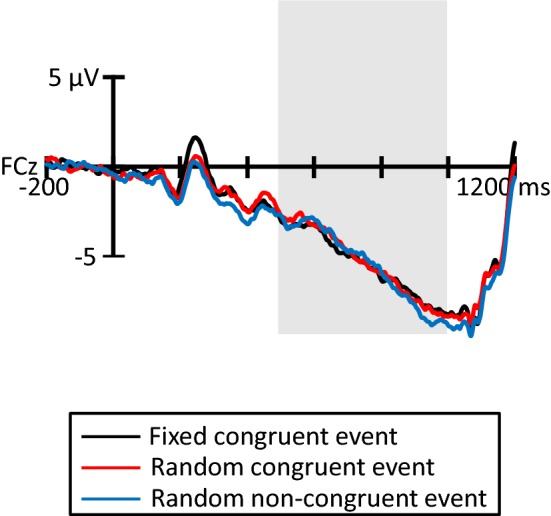
Grand average ERP waveforms for each congruent event at FCz (*N* = 14). The gray area indicates the time range for CNV (500–1000 ms)

Figure 6 illustrates the grand average CNV elicited in all trials at FCz, where the CNV was elicited at maximum amplitude. The gray area indicates the time range of CNV (500–1000 ms). The ANOVA showed that the CNV revealed no significant difference among the fixed congruent event, random congruent event, and random non-congruent event (*F*(2, 26) = 0.07, *p* > 0.10).

## Discussion

The present study aimed to investigate the influence of a relationship between self-induced action and intervening events on expectations of a final result. Previous studies reported that ERP amplitudes about 100 ms after a stimulus decreased due to sensory attenuation (e.g., Bäß et al. 2008; Horváth 2015; Martikainen et al. 2005; Palmer et al. 2016; Schafer and Marcus 1973). Moreover, an external somatosensory stimulus, without self-induced action, elicited P1 in a condition similar to the present experimental condition and that P1 amplitude did not differ between high-probability stimuli and low-probability stimuli (Kimura and Katayama 2015, a). Therefore, P1 amplitude was examined as an index of sensory attenuation in this study.

Our results showed that P1 amplitudes elicited by high-probability somatosensory stimuli decreased in fixed congruent events. In this study, the delay between self-induced action and final result was 4000 ms, which is longer than in typical previous studies. However, a previous study reported that participants were able to predict the feedback (i.e., final result) when intervening stimuli were presented regularly between a self-induced action and feedback, even if the delay was 6000 ms (Kimura and Kimura 2016). They reported an effect of temporal regularity of stimuli presented between actions and results on the processing of results caused by self-actions. In our study, stimuli (i.e., intervening events) were presented between self-actions and final events, and regularity between self-actions and intervening events (i.e., congruency) influenced the prediction of final events. Therefore, the results of the present study showed that the final result can be predicted by this regularly intervening event and that sensory attenuation occurs in fixed congruent events. Regarding comparisons between the present study and typical action-based prediction experiments, further research will be needed.

By contrast, P1 amplitudes did not decrease in random congruent events and random non-congruent events. In the present study, spatial congruency existed between the intervening event and final result in all congruent events such that high-probability somatosensory stimuli were presented on the same side as the white circles in all congruent events. Therefore, it is assumed that the P1 amplitudes in the random congruent events and random non-congruent events should decrease by the same amount as in the fixed congruent events if congruency of a final result and an intervening event is important for predicting the final result. Moreover, although the white circles were presented on the same side on which a key was pressed in random congruent events, which was the same in the fixed congruent events, P1 amplitude decreased only in the fixed congruent condition. The difference between these trials was only the presence or absence of congruency of an intervening event and self-induced action; white circles flashed invariably on the same side on which the button was pressed only in fixed congruent events. Therefore, the results of this study indicate that congruency of an intervening event with self-induced action facilitates the prediction of final results. More importantly, this congruency was irrelevant information for the simple reaction time task in response to the somatosensory stimulus because high-probability somatosensory stimuli were presented not on the same side on which the key was pressed but on the same side on which the white circles were presented in all congruent events. In other words, this result suggests that prediction of the final result was influenced by the predictability of congruency between self-induced action and event, even though this congruency is irrelevant for the ongoing task.

In addition, our results showed that whereas P3 was elicited by low-probability somatosensory stimuli, the amplitudes did not differ among congruent events. Previous studies reported that P3a elicited by task-irrelevant deviant stimuli (i.e., infrequent non-target stimuli) was enhanced by the stimuli generated by self-induced action compared to the stimuli generated by exterior events. By contrast, the P3b elicited by task-relevant low-probability stimuli (i.e., infrequent target stimuli) did not differ between those generated by self-induced action and those generated externally (e.g., von Carlowitz-Ghori et al. 2011; Nittono 2006). P3a and P3b are subcomponents of P3. It is known that P3a is elicited by non-target deviant stimuli or novel stimuli and P3b is elicited by infrequent target stimuli (e.g., Katayama and Polich 1998). In the present study, participants performed simple reaction time tasks in response to all somatosensory stimuli. In other words, all somatosensory stimuli were target stimuli; therefore, it is possible that the P3b elicited by low-probability target somatosensory stimuli was not influenced by self-induced action. The results of the present study may indicate that congruency of an intervening event and self-induced action did not influence deviations from predictions elicited by task-relevant information as in previous studies.

The results showed that the RTs to low-probability stimuli were longer than those to high-probability stimuli, but RTs did not differ among congruent events. Moreover, the CNV which was the ERP in conjunction with the temporal prediction (e.g., Walter et al. 1964) did not differ across the congruent events. These results suggest that the participants created temporal expectations about the timing of the subsequent somatosensory stimuli in all congruent events.

According to the internal forward model, self-induced action facilitates prediction of self-induced events because an internal monitoring system based on planning of self-induced action, copying of motor commands, and sensory feedback resulting from self-induced action is used in self-induced action (e.g., Blakemore et al. 1999; Wolpert 1997; Wolpert et al. 1995). In addition, recent studies have reported that temporal predictability influences prediction of results whether temporal prediction derives from self-induced actions or external ones (e.g., Baess et al. 2011; Horváth 2015; Hughes et al. 2013; Lange 2011). In the results of our study, all congruent events had similar temporal predictability because the results of RT and CNV did not differ among congruent events, and the difference between these congruent events was only the presence or absence of congruency between self-induced action and intervening events. Thus, our results indicate that congruency of intervening events and self-induced action influences prediction of self-induced events in addition to temporal prediction.

Finally, several points about this result need to be considered. At first, the experimental paradigm included a spatial factor between the self-induced action and intervening event, and it is possible that spatial attention is elicited by this paradigm (e.g., Horváth 2015; Hughes and Waszak 2011). Previous studies reported a different effect of attention on sensory attenuation. For example, some studies suggested that sensory attenuation effects did not change at different attention levels and was independent of attention (e.g., Timm et al. 2013), while other studies reported an effect of attention on sensory attenuation (e.g., Cao and Gross 2015; Jones et al. 2013). One explanation for the sensory attenuation is an internal forward model in which action and prediction are related (e.g., Blakemore et al. 1999; Wolpert 1997; Wolpert et al. 1995), and this relationship could be considered using predictive coding (e.g., Friston 2012). In predictive coding, prediction is calculated by prediction error, and some studies have reported that attention influences prediction (e.g., Schröger et al. 2015). Therefore, it is possible that our results were influenced by it if spatial attention was elicited by our paradigm. Presenting a visual stimulus invariably at an equal distance from the side of self-induced left and right action in fixed congruent trials might make it possible to separate the spatial factor when this point is examined (e.g., Kimura and Katayama 2015 Exp. 1), because the visual stimulus does not include a spatial factor in this condition. Moreover, it might also be useful that the visual stimulus is presented invariably on the opposite side of the self-induced action in fixed congruent trials (e.g., Kimura and Katayama 2015 Exp. 2), because the spatial factor of the visual stimulus and final event does not match in this condition. If the spatial factor facilitates the prediction of the final event, it is possible that predictive coding is facilitated in the condition with a spatial factor, and that P1 amplitude under this condition would decrease more than under the condition of not having or not matching the spatial factor. Moreover, it is possible that the prediction error for the stimulus that deviates from the spatial factor increases under this condition, and that P3 amplitude is larger than under the condition of not having or not matching the spatial factor (e.g., Kimura and Katayama 2015). Regarding the relationship between spatial attention and predictive coding, further study will be needed. Second, it is possible that this result can be explained by not only the congruency of an intervening event and self-induced action, but also self-action-based prediction, because final events under the fixed congruent condition could be predicted based on the side on which actions occur as well as the intervening event. This point might be verified by examining a modified condition from a previous study (Kimura and Katayama 2015 Exp. 2) where there is congruency between the self-action and the final event, such as self-action (right), intervening event (left), and high-probability final event (right).

In summary, the present study revealed that congruency of an intervening event and self-induced action decreases the P1 amplitude elicited in response to the final result, indicating that this congruency facilitates prediction of the final result, even though the congruency is irrelevant for the ongoing task. The present study expands our understanding of prediction of self-induced events and sensory attenuation.
